# Arthritis–diabetes comorbidity and functional recovery in older adults: a longitudinal cohort study using the Health and Retirement Study

**DOI:** 10.21203/rs.3.rs-10606280/v1

**Published:** 2026-09-10

**Authors:** Jinkui Guo, Zhi Li, Weihua Yang

**Affiliations:** The 95 Hospital of Putian; The 95 Hospital of Putian; The 95 Hospital of Putian

**Keywords:** arthritis, diabetes, disability, functional recovery, multimorbidity, mortality

## Abstract

**Background:**

Arthritis and diabetes commonly coexist in older adults, but recovery after disability has received less attention than disability onset. We examined whether coexisting diabetes was associated with disability onset, functional recovery, state-specific transitions, and mortality among older adults with arthritis.

**Methods:**

This longitudinal cohort study used biennial Health and Retirement Study data from 2000 to 2022. Consecutive interview pairs contributed person-wave observations for adults aged 65 years or older. Time-updated arthritis and diabetes status defined four exposure groups. Functional status was classified as complete independence, instrumental activities of daily living (IADL) limitation, or activities of daily living (ADL) disability. Pooled multinomial logistic models estimated standardized 2-year probabilities with participant-clustered robust covariance. The primary comparison was arthritis–diabetes comorbidity versus arthritis only.

**Results:**

The disability-onset analysis included 69,075 person-waves from 17,540 participants, and the recovery analysis included 23,725 person-waves from 10,066 participants. Comorbidity was associated with a higher probability of incident disability (17.1% vs 15.8%; risk difference [RD], + 1.30 percentage points; 95% confidence interval [CI], + 0.41 to + 2.22). Recovery to complete independence was less likely with comorbidity (19.9% vs 23.3%; RD, −3.42 percentage points; 95% CI, −4.72 to −2.10). Among observations beginning with ADL disability, any functional improvement was also less likely (23.0% vs 27.7%; RD, −4.71 percentage points; 95% CI, −6.43 to −2.99).

**Conclusions:**

Coexisting diabetes was associated with a modest increase in disability onset but clearer reductions in recovery and functional improvement, together with higher mortality, among older adults with arthritis. These findings emphasize functional prognosis after disability in addition to prevention of disability onset.

## Background

Disability in later life is not always permanent. Older adults may move between independence, functional limitation, more severe disability, and death [1–3]. Activities of daily living (ADL) and instrumental activities of daily living (IADL) capture different levels of functional dependence [4, 5]. Recovery is clinically important because regaining independence can preserve autonomy and reduce the need for assistance.

Arthritis and diabetes can both impair function, but they do so through partly different pathways. In arthritis, pain, stiffness, and restricted mobility contribute to functional decline [6, 7]. In diabetes, vascular, metabolic, and neuromuscular complications can lead to mobility loss and disability [8]. When the conditions coexist, these problems may further reduce the ability to recover from a functional setback. Recent multicohort evidence also suggests that diabetes and arthritis may influence each other over time [9].

Previous HRS studies have shown that disease combinations involving arthritis or diabetes are common and are associated with greater ADL and IADL burden [10, 11]. A CHARLS study examined disability onset, recovery of independence, and death across broad multimorbidity patterns [12], and other longitudinal research has linked multimorbidity patterns with disability [13]. These studies do not directly answer whether diabetes changes the functional course of older adults who already have arthritis. In particular, little is known about recovery once disability has occurred in this group.

We used repeated HRS observations from 2000 through 2022 to examine the association of time-updated arthritis–diabetes status with 2-year functional transitions and mortality in adults aged 65 years or older. The primary comparison was between participants with both conditions and those with arthritis alone. We examined disability onset among participants who were initially independent, recovery among those with IADL or ADL difficulty, transitions among the three functional states, and death. Recovery was also assessed separately for recent-onset and persistent disability.

## Methods

### Study design and data source

We conducted a longitudinal secondary analysis of the Health and Retirement Study (HRS), a national panel study of U.S. adults older than 50 years and their spouses [14, 15]. The primary analysis used the RAND HRS Longitudinal File 2022 (Version 1; including the 2022 Early Release) [16] and the Health and Retirement Study 2022 Tracker Final, Version 1.0 [17]. RAND records were linked to the Tracker by participant identifier for mortality information. Public HRS Core files from 2000–2022 were used only for variable validation and the prespecified contemporaneous-disease sensitivity analysis [18]. Reporting followed the Strengthening the Reporting of Observational Studies in Epidemiology (STROBE) recommendations [19].

This analysis uses Early Release data from the Health and Retirement Study, RAND HRS Longitudinal File 2022 (Version 1), sponsored by the National Institute on Aging (grant number NIA U01AG009740) and conducted by the University of Michigan. These data have not been cleaned and may contain errors that will be corrected in the Final Release version of the dataset.

The HRS protocol was approved by the University of Michigan Institutional Review Board, and all participants provided informed consent. The present study used deidentified secondary data and involved no new recruitment or participant contact. No additional consent or ethics approval was required.

### Construction of the adjacent-wave cohort

We organized the data as rolling pairs of consecutive HRS interviews. Exposure, covariates, and functional state were measured at each baseline wave t, and functional state or death was assessed at the next wave t + 1. Participants could contribute more than one person-wave when they met the eligibility criteria at several baseline waves. This approach allowed disease status and covariates to be updated over time.

A person-wave was eligible when the participant was aged 65 years or older and had observed arthritis status, diabetes status, and baseline functional state. The starting dataset contained 497,574 candidate adjacent-wave observations. After the eligibility criteria were applied, 115,462 person-waves from 23,716 participants remained. Individual models excluded observations with missing required covariates or missing next-wave functional status among survivors. Death was retained as an outcome rather than treated as missing follow-up. Figure 1 presents the construction of the analytic risk sets.

### Arthritis and diabetes status

At each baseline wave, arthritis and diabetes were identified from reports of a physician diagnosis of arthritis or rheumatism and diabetes or high blood sugar, respectively. The arthritis measure did not distinguish osteoarthritis from rheumatoid arthritis and did not include anatomical site or disease severity. The two indicators defined four time-varying groups: neither condition, arthritis only, diabetes only, and arthritis–diabetes comorbidity. Arthritis only was the prespecified reference group because the study asked whether diabetes was associated with additional functional disadvantage among older adults with arthritis.

### Functional states and outcomes

Functional difficulty was assessed in six ADL tasks (bathing or showering, dressing, eating, getting in or out of bed, walking across a room, and using the toilet) and five IADL tasks (preparing hot meals, shopping for groceries, using the telephone, taking medications, and managing money) [4, 5]. Any difficulty recorded in the harmonized HRS indicator counted as difficulty with that task. Complete information for all tasks in a domain was required for the primary classification.

Participants were assigned to one of three mutually exclusive functional states at each wave. Complete independence meant no difficulty with any ADL or IADL task. IADL limitation meant difficulty with at least one IADL task and no ADL difficulty. ADL disability meant difficulty with at least one ADL task, regardless of IADL status. This hierarchy distinguishes limitations in basic self-care from IADL difficulty without assuming that everyone follows the same sequence of decline.

The disability-onset analysis included person-waves that began in complete independence. At the next wave, participants could remain independent, develop IADL or ADL disability, or die. The recovery analysis included person-waves that began with IADL limitation or ADL disability. At the next wave, participants could remain disabled, return to complete independence, or die. Recovery required complete ADL and IADL independence.

We also fitted separate multinomial models for observations beginning in complete independence, IADL limitation, and ADL disability. The possible next-wave outcomes were complete independence, IADL limitation, ADL disability, and death. Because HRS interviews are approximately two years apart, these outcomes describe the state observed at the next interview and do not capture all changes that may have occurred between interviews.

### Covariates

Covariates were selected before analysis and included age; sex; race/ethnicity; education; marital status; household assets transformed as sign(x) × log(1 + |x|); body mass index; current smoking; RAND binary ever alcohol use; other chronic-condition count (hypertension, heart disease, stroke, lung disease, and cancer, excluding arthritis and diabetes); RAND total cognition score (0–35, with mode-adapted scores in later waves) [20]; RAND 8-item CES-D summary score (0–8); hospital stay since the prior interview (approximately two years); and survey wave/year. Age was modeled with a cubic B-spline with 4 degrees of freedom using Patsy bs(age, df = 4). Recovery models additionally included baseline ADL and IADL difficulty counts. Primary models were complete-case analyses. Because cognition and depressive-symptom scores were structurally unavailable for proxy interviews, all complete cases contributing to the primary models were self-interviews.

### Statistical analysis

Baseline characteristics were summarized at each participant’s first eligible observation for the four arthritis–diabetes groups. Continuous variables are reported as means with standard deviations or medians with interquartile ranges, and categorical variables as counts and percentages. Absolute standardized mean differences (SMDs) described differences between the comorbidity and arthritis-only groups.

Pooled multinomial logistic regression models were fitted within each risk set. Standard errors were clustered by participant because individuals could contribute repeated person-waves. Arthritis only was the reference exposure. Standardized probabilities were obtained by predicting each outcome for every observation under each exposure category while retaining observed covariate values, then averaging predictions across the complete-case analytic sample [21]. Risk differences were comorbidity minus arthritis only. For the primary and extension models, 95% confidence intervals used the 2.5th and 97.5th percentiles from 20,000 joint multivariate-normal parameter draws based on participant-clustered robust covariance. All outcomes and composites from a model used the same fitted object and parameter draws.

Primary models used complete-case observations and were not survey weighted. Respondent weights were not consistently available across waves, and requiring them would have substantially reduced the longitudinal sample. The estimates therefore describe adjusted associations in the analytic HRS sample and should not be read as nationally representative absolute risks. Survey-wave indicators accounted for differences across calendar years.

In prespecified extension analyses, the recovery risk set was divided into recent-onset disability and persistent disability. Recent-onset disability was defined as independence at wave t − 1 followed by disability at wave t; persistent disability was defined as disability at both waves t − 1 and t. For observations beginning with ADL disability, any functional improvement included transition to complete independence or IADL limitation. We also fitted proxy-compatible reduced-adjustment sensitivity models that omitted cognition and CES-D: among self-interviews and among all self and proxy interviews (the latter additionally adjusted for proxy status).

Sensitivity analyses omitted the count of other chronic conditions; excluded observations with cognition scores of 6 or lower; treated the raw HRS ‘does not do’ response as either missing or difficulty; applied non-stabilized inverse-probability weights for observed non-death follow-up (capped at 10) [22]; required two consecutive positive reports to confirm arthritis or diabetes; repeated the analyses using contemporaneous raw HRS Core disease variables; and used proxy-compatible reduced-adjustment models.

### Use of generative AI-assisted technologies

During manuscript preparation, the authors used ChatGPT (OpenAI) for language editing and submission-formatting support. The authors reviewed and edited all output and take full responsibility for the content of the manuscript.

## Results

### Analytic samples

The initial dataset contained 497,574 candidate adjacent-wave person-waves. A total of 115,462 observations from 23,716 participants met the age, exposure, and baseline-function criteria. The baseline risk sets included 80,685 independent person-waves, 7,782 person-waves with IADL limitation, and 26,995 person-waves with ADL disability. After complete-case and next-wave outcome restrictions, the disability-onset model included 69,075 person-waves from 17,540 participants. The IADL-origin and ADL-origin transition models included 5,643 person-waves from 3,855 participants and 18,082 person-waves from 8,151 participants, respectively. The two disability groups together contributed 23,725 person-waves from 10,066 participants to the overall recovery analysis (Fig. 1; see Additional file 1: Supplementary Table S1).

### Baseline characteristics

At the first eligible observation, 8,003 participants had neither condition, 10,766 had arthritis only, 1,638 had diabetes only, and 3,309 had arthritis–diabetes comorbidity (Table 1). Compared with the arthritis-only group, participants with comorbidity were slightly younger on average (69.13 vs 70.96 years), had a higher body mass index (31.04 vs 27.31 kg/m^2^; absolute SMD, 0.61), and had more other chronic conditions (median, 2 vs 1; SMD, 0.50). They also had lower household assets and cognition scores, more depressive symptoms, more recent hospitalization, and higher baseline ADL and IADL difficulty counts.

### Disability onset from complete independence

Among observations beginning in complete independence, the standardized probability of remaining independent at the next wave was 76.4% (95% CI, 75.4%–77.2%) with comorbidity and 79.4% (95% CI, 78.9%–79.9%) with arthritis only (Table 2 and Fig. 2). The probability of developing disability was 17.1% and 15.8%, respectively, corresponding to an RD of + 1.30 percentage points (95% CI, + 0.41 to + 2.22). The probability of death was 6.6% and 4.8%, respectively (RD, + 1.76 percentage points; 95% CI, + 1.24 to + 2.30).

### Recovery from disability

Among observations beginning with IADL limitation or ADL disability, the adjusted probability of recovery to complete independence was 19.9% with comorbidity and 23.3% with arthritis only (RD, −3.42 percentage points; 95% CI, −4.72 to −2.10) (Table 2 and Fig. 2). Death occurred in 19.0% and 15.5%, respectively (RD, + 3.48 percentage points; 95% CI, + 2.34 to + 4.65).

### State-specific functional transitions

The state-specific models identified the transitions that contributed to the overall results (Fig. 3; see Additional file 1: Supplementary Table S2 and Additional file 2: Supplementary Fig. S1). Among observations beginning in complete independence, comorbidity was not clearly associated with isolated IADL limitation (RD, + 0.18 percentage points; 95% CI, − 0.32 to + 0.70). It was associated with a higher probability of ADL disability at the next wave (RD, + 1.10 percentage points; 95% CI, + 0.34 to + 1.89) and a higher probability of death (RD, + 1.76 percentage points; 95% CI, + 1.25 to + 2.32). This result does not show that participants bypassed an IADL stage because functional status could change between the biennial interviews.

Among observations beginning with IADL limitation, comorbidity was associated with a lower probability of returning to complete independence (RD, − 4.67 percentage points; 95% CI, − 7.83 to − 1.47) and a higher probability of death (RD, + 3.31 percentage points; 95% CI, + 0.77 to + 5.92). The estimate for progression to ADL disability was less precise and included no difference (RD, + 2.67 percentage points; 95% CI, − 0.69 to + 6.01).

Among observations beginning with ADL disability, comorbidity was associated with lower probabilities of returning to complete independence (RD, −3.69 percentage points; 95% CI, −5.20 to −2.13) and improving to IADL limitation (RD, −1.02 percentage points; 95% CI, −1.88 to −0.15). Death was more likely (RD, + 3.70 percentage points; 95% CI, + 2.41 to + 5.02).

### Disability history and functional-improvement extensions

The association between comorbidity and lower recovery was present in both disability-history groups (see Additional file 3: Supplementary Fig. S2 and Additional file 1: Supplementary Table S3). Among observations with recent-onset disability, recovery to independence had an RD of −3.52 percentage points (95% CI, −5.99 to −1.06). Among observations with persistent disability, the recovery RD was – 2.06 percentage points (95% CI, −3.39 to −0.69), and the mortality RD was + 4.44 percentage points (95% CI, + 2.79 to + 6.06).

Among observations beginning with ADL disability, any functional improvement was defined as return to complete independence or improvement to IADL limitation. Improvement occurred in 23.0% with comorbidity and 27.7% with arthritis only. The RD was – 4.71 percentage points (95% CI, −6.43 to −2.99).

### Sensitivity analyses

Across the prespecified disease-definition, covariate-specification, attrition-weighting, and proxy-compatible reduced-adjustment analyses, the direction of the primary comparison was unchanged (see Additional file 1: Supplementary Table S4). The proxy-inclusive reduced-adjustment models supported higher disability onset and lower recovery with comorbidity than with arthritis only.

## Discussion

In this longitudinal HRS analysis, diabetes was associated with only a modest increase in new disability among participants with arthritis who were initially independent. The differences were clearer after disability had occurred. Participants with both conditions were less likely to regain independence or improve from ADL disability and were more likely to die than participants with arthritis alone. Lower recovery was observed in both recent-onset and persistent disability, and the estimates were stable across the sensitivity analyses.

Recovery warrants separate study because disability in later life can improve as well as worsen. Previous longitudinal studies show that many older adults regain independence, especially after recent-onset disability, while prolonged disability and frailty reduce the likelihood of recovery [23–26]. Comorbidity, frailty, and disability overlap but describe different aspects of health [27]. Hospitalization and the final phase of life can also move functional trajectories toward persistent disability or death [28, 29]. An analysis limited to disability onset would miss much of this variation.

The lower recovery observed in participants with both conditions is biologically plausible. Arthritis-related pain, stiffness, and restricted mobility may limit activity and participation in rehabilitation [6, 7]. Diabetes may add neuropathy, vascular disease, visual impairment, obesity, and poorer muscle quality. Glycemic burden and related comorbidities may further reduce functional reserve [8, 30, 31]. Recent multicohort findings also point to inflammatory, glycemic, and strength-related links between diabetes and arthritis [9]. These mechanisms were not measured directly in HRS and should be regarded as possible explanations rather than demonstrated pathways.

Previous research has generally examined multimorbidity in broader categories. HRS analyses have shown that disease combinations involving arthritis or diabetes are common and are associated with poorer function [10, 11]. Zhou and colleagues examined disability onset, recovery, and mortality across broad multimorbidity patterns in CHARLS [12]. Other longitudinal studies have linked multimorbidity with functional decline and survival [13, 32], and diabetes-related disease combinations have also been associated with disability [33]. A recent meta-analysis reported a similar association between multimorbidity and disability [34]. We addressed a narrower clinical question by comparing older adults with arthritis according to whether diabetes was also present. Updating disease status at each wave and separating onset, recovery, partial improvement, specific transitions, and death showed that the main difference concerned recovery after disability rather than disability onset alone.

The absolute difference in disability onset was modest. Over one survey interval, comorbidity was associated with about 1.3 additional disability events per 100 initially independent person-waves. Among observations beginning with disability, there were about 3.4 fewer complete recoveries per 100 person-waves. Among those beginning with ADL disability, any functional improvement was 4.7 percentage points less common. The onset and recovery analyses involved different risk sets, so these estimates should not be compared as measures of the same outcome. The association with recovery was nevertheless more consistent than the association with new disability.

The individual transition estimates showed where these differences arose. Among participants who were independent, comorbidity was associated with ADL disability and death at the next interview but not clearly with isolated IADL limitation. The two-year interval prevents a firm conclusion about the sequence of intermediate states. Among participants with IADL limitation, comorbidity was associated with lower recovery and higher mortality, while progression to ADL disability remained uncertain. Among participants with ADL disability, both complete recovery and partial improvement were less common. The results did not indicate a uniform increase in every worsening transition. Lower improvement and higher mortality were the recurring differences.

Treating death as a separate outcome was important. In the overall recovery risk set, the probability of remaining disabled was almost the same in the comorbidity and arthritis-only groups, but recovery was less common and death was more common with comorbidity. A binary analysis that combined death with persistent disability, or removed deaths as ordinary missing follow-up, would have obscured this difference. Previous transition studies also show that recovery and mortality are closely connected in late-life functional change [2, 3].

For clinical practice, the results draw attention to follow-up after functional decline in older adults with both arthritis and diabetes. Monitoring mobility, pain, depressive symptoms, obesity, diabetes-related complications, and functional needs after hospitalization may be relevant. The study cannot determine whether tighter glycemic control, a particular arthritis treatment, or any specific intervention would improve recovery.

More than two decades of repeated HRS interviews allowed disease status to be updated at each wave. Separate risk sets for onset and recovery, standardized absolute probabilities, and treatment of death as an outcome helped distinguish new disability from failure to recover. Estimates were similar across several sensitivity analyses.

The study has important limitations. Arthritis and diabetes were based on reports of physician diagnosis, and consistent information on arthritis subtype, disease severity, diabetes complications, and treatment was not available across waves. Functional status was self- or proxy-reported at roughly two-year intervals, so shorter episodes of decline or recovery may have been missed. Because cognition and depressive-symptom scores were structurally unavailable for proxy interviews, the fully adjusted primary analyses were restricted to self interviews and may underrepresent participants with more severe cognitive or functional impairment. Proxy-inclusive reduced-adjustment analyses were directionally consistent, but residual selection bias remains possible. Residual confounding and selection related to other missing data also remain possible. Because the models were unweighted and observational, the estimates apply to the analytic HRS sample and should not be interpreted as causal effects or nationally representative absolute risks.

## Conclusions

Among adults aged 65 years or older with arthritis, coexisting diabetes was associated with a modest increase in new disability, lower probabilities of complete recovery and partial functional improvement after disability, and higher mortality. The results place particular emphasis on recovery after functional decline, in addition to prevention of disability onset.

## Supplementary Material

This is a list of supplementary files associated with this preprint. Click to download.

- Additionalfile1.docx
- SupplementaryFigS1.tiff
- SupplementaryFigS2.tiff

## Figures and Tables

**Figure 1:**
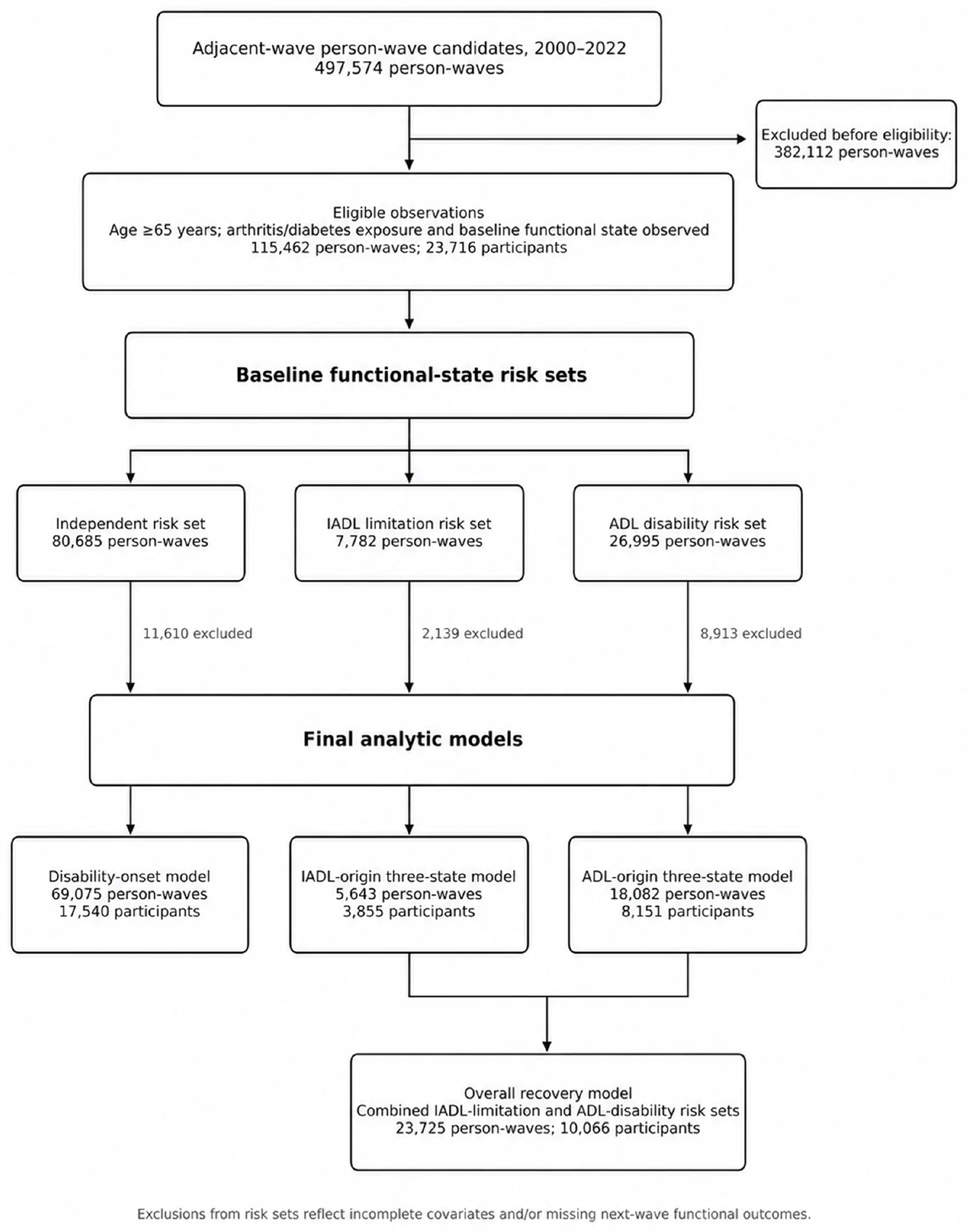
Study flow and analytic risk sets. ADL, activities of daily living; IADL, instrumental activities of daily living; HRS, Health and Retirement Study.

**Figure 2:**
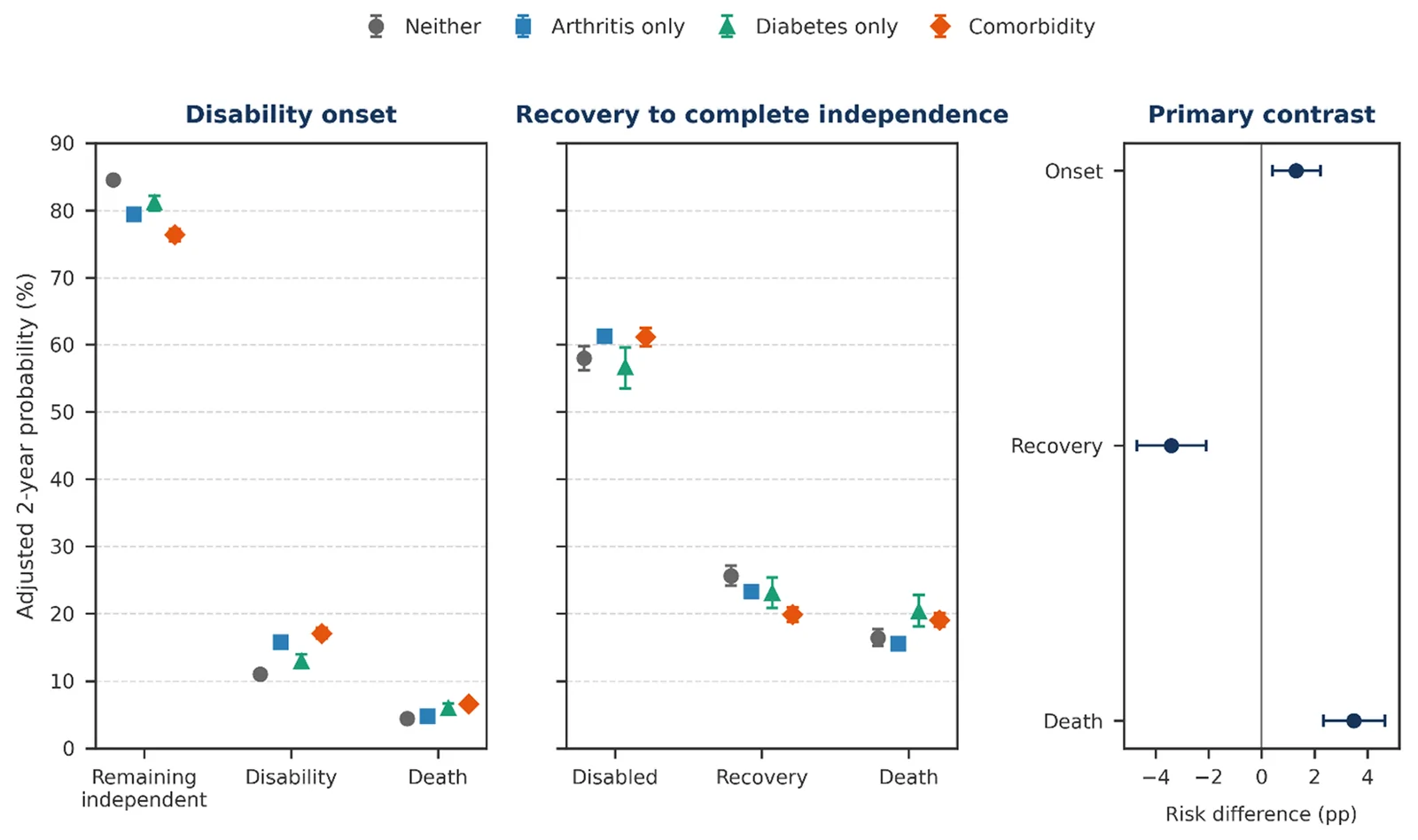
Disability onset, recovery, and death by disease status. Adjusted 2-year probabilities were standardized from pooled multinomial models. Error bars indicate 95% confidence intervals. Colors and marker shapes identify exposure groups. The primary-contrast panel displays comorbidity-minus-arthritis-only risk differences in percentage points.

**Figure 3:**
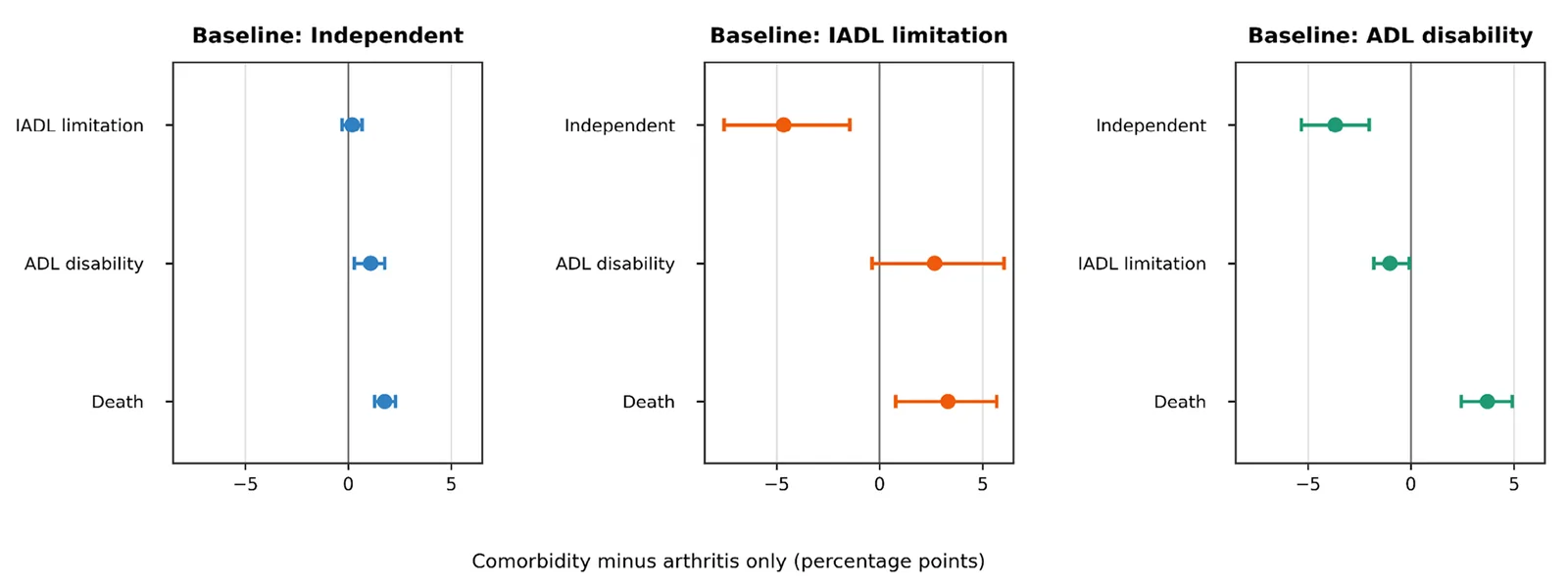
Risk differences for selected state-specific functional transitions. Estimates compare arthritis–diabetes comorbidity with arthritis only. Positive values indicate higher probabilities with comorbidity and negative values indicate lower probabilities. Error bars indicate 95% confidence intervals.

**Table 1:** Baseline characteristics by arthritis and diabetes status *First eligible observation for each participant; adults aged 65 years or older, HRS 2000–2022*.

Characteristic	Neither	Arthritis only	Diabetes only	Comorbidity	Absolute SMD
Participants, n	8003	10766	1638	3309	
Age, years, mean (SD)	70.06 (6.67)	70.96 (7.55)	69.03 (5.94)	69.13 (6.15)	0.27
ADL difficulty count, median [IQR]	0.00 [0.00, 0.00]	0.00 [0.00, 0.00]	0.00 [0.00, 0.00]	0.00 [0.00, 1.00]	0.24
IADL difficulty count, median [IQR]	0.00 [0.00, 0.00]	0.00 [0.00, 0.00]	0.00 [0.00, 0.00]	0.00 [0.00, 1.00]	0.16
Household assets, signed log scale, median [IQR]	12.30 [11.10, 13.25]	11.93 [10.55, 12.95]	11.75 [10.41, 12.78]	11.25 [8.85, 12.38]	0.29
Body mass index, kg/m^2^, mean (SD)	26.17 (4.64)	27.31 (5.56)	28.74 (5.27)	31.04 (6.66)	0.61
Other chronic condition count, median [IQR]	1.00 [0.00, 1.00]	1.00 [0.00, 2.00]	1.00 [1.00, 2.00]	2.00 [1.00, 2.00]	0.50
Cognition, mean (SD)	22.86 (5.10)	22.35 (5.13)	21.55 (5.21)	21.03 (5.18)	0.25
Depressive symptoms (CES-D), median [IQR]	0.00 [0.00, 1.00]	1.00 [0.00, 2.00]	1.00 [0.00, 2.00]	1.00 [0.00, 3.00]	0.25
**Sex, n (%)**					
Male	4003/8003 (50.0%)	3930/10766 (36.5%)	938/1638 (57.3%)	1348/3309 (40.7%)	0.09
Female	4000/8003 (50.0%)	6836/10766 (63.5%)	700/1638 (42.7%)	1961/3309 (59.3%)	0.09
**Race/ethnicity, n (%)**					
White	6536/7993 (81.8%)	8709/10758 (81.0%)	1117/1635 (68.3%)	2207/3304 (66.8%)	0.33
Black/African American	1029/7993 (12.9%)	1613/10758 (15.0%)	357/1635 (21.8%)	831/3304 (25.2%)	0.26
Other	428/7993 (5.4%)	436/10758 (4.1%)	161/1635 (9.8%)	266/3304 (8.1%)	0.17
**Education, n (%)**					
Less than high school	1699/8001 (21.2%)	2854/10763 (26.5%)	498/1638 (30.4%)	1121/3309 (33.9%)	0.16
GED	292/8001 (3.6%)	489/10763 (4.5%)	67/1638 (4.1%)	180/3309 (5.4%)	0.04
High school graduate	2373/8001 (29.7%)	3318/10763 (30.8%)	430/1638 (26.3%)	926/3309 (28.0%)	0.06
Some college	1695/8001 (21.2%)	2241/10763 (20.8%)	331/1638 (20.2%)	664/3309 (20.1%)	0.02
College or above	1942/8001 (24.3%)	1861/10763 (17.3%)	312/1638 (19.0%)	418/3309 (12.6%)	0.13
**Marital status, n (%)**					
Married	5099/7997 (63.8%)	6180/10759 (57.4%)	1046/1636 (63.9%)	1802/3306 (54.5%)	0.06
Married, spouse absent	65/7997 (0.8%)	81/10759 (0.8%)	21/1636 (1.3%)	39/3306 (1.2%)	0.04
Partnered	284/7997 (3.6%)	350/10759 (3.3%)	74/1636 (4.5%)	130/3306 (3.9%)	0.04
Separated	106/7997 (1.3%)	146/10759 (1.4%)	34/1636 (2.1%)	78/3306 (2.4%)	0.07
Divorced	715/7997 (8.9%)	1054/10759 (9.8%)	157/1636 (9.6%)	369/3306 (11.2%)	0.04
Separated or divorced	0/7997 (0.0%)	2/10759 (0.0%)	0/1636 (0.0%)	0/3306 (0.0%)	0.02
Widowed	1465/7997 (18.3%)	2598/10759 (24.1%)	258/1636 (15.8%)	747/3306 (22.6%)	0.04
Never married	263/7997 (3.3%)	348/10759 (3.2%)	46/1636 (2.8%)	141/3306 (4.3%)	0.05
**Current smoking, n (%)**					
No	6925/7980 (86.8%)	9404/10741 (87.6%)	1459/1632 (89.4%)	2936/3293 (89.2%)	0.05
Yes	1055/7980 (13.2%)	1337/10741 (12.4%)	173/1632 (10.6%)	357/3293 (10.8%)	0.05
**Alcohol use, n (%)**					
No	3702/8000 (46.3%)	5692/10763 (52.9%)	979/1637 (59.8%)	2201/3309 (66.5%)	0.28
Yes	4298/8000 (53.7%)	5071/10763 (47.1%)	658/1637 (40.2%)	1108/3309 (33.5%)	0.28
**Recent hospital stay, n (%)**					
No	6353/7987 (79.5%)	7521/10741 (70.0%)	1170/1634 (71.6%)	1993/3292 (60.5%)	0.20
Yes	1634/7987 (20.5%)	3220/10741 (30.0%)	464/1634 (28.4%)	1299/3292 (39.5%)	0.20
**Proxy interview, n (%)**					
Self interview	7333/8003 (91.6%)	9689/10766 (90.0%)	1480/1638 (90.4%)	3000/3309 (90.7%)	0.02
Proxy interview	670/8003 (8.4%)	1077/10766 (10.0%)	158/1638 (9.6%)	309/3309 (9.3%)	0.02

Note. Values are mean (SD), median [IQR], or n (%), as indicated. Denominators may vary because of item-level missing data. Absolute SMDs compare the comorbidity group with the arthritis-only group.

ADL, activities of daily living; IADL, instrumental activities of daily living; CES-D, Center for Epidemiologic Studies Depression scale.

**Table 2:** Adjusted 2-year probabilities of disability onset, recovery, and death *Estimates were standardized from pooled multinomial models with participant-clustered robust covariance*.

Next-wave outcome	Neither, % (95% CI)	Arthritis only, % (95% CI)	Diabetes only, % (95% CI)	Comorbidity, % (95% CI)	Risk difference, percentage points (95% CI)
Disability-onset risk set (69075 person-waves; 17540 participants)					
Remaining independent	84.5 (84.0–85.0)	79.4 (78.9–79.9)	81.2 (80.0–82.2)	76.4 (75.4–77.2)	−3.07 (−4.07 to −2.08)
Disability	11.0 (10.6–11.5)	15.8 (15.3–16.2)	12.9 (11.9–13.9)	17.1 (16.3–17.9)	+ 1.30 (+ 0.41 to + 2.22)
Death	4.4 (4.2–4.7)	4.8 (4.6–5.1)	6.0 (5.4–6.7)	6.6 (6.1–7.1)	+ 1.76 (+ 1.24 to + 2.30)
Functional-recovery risk set (23725 person-waves; 10066 participants)					
Remaining disabled	58.0 (56.1–59.7)	61.2 (60.3–62.0)	56.6 (53.5–59.5)	61.1 (59.7–62.4)	−0.07 (−1.71 to + 1.54)
Recovery to complete independence	25.6 (24.2–27.1)	23.3 (22.6–24.0)	23.1 (20.8–25.4)	19.9 (18.8–21.0)	−3.42 (−4.72 to −2.10)
Death	16.4 (15.3–17.7)	15.5 (15.0–16.1)	20.4 (18.1–22.8)	19.0 (18.1–20.1)	+ 3.48 (+ 2.34 to + 4.65)

Note. Risk differences compare comorbidity with arthritis only (comorbidity minus arthritis only). Death was modeled as a separate outcome; recovery was defined as return to complete ADL and IADL independence. Models were restricted to complete self-interview observations because cognition and CES-D scores were structurally unavailable for proxy interviews. Models adjusted for age, sex, race/ethnicity, education, marital status, household assets, body mass index, smoking, ever alcohol use, other chronic-condition count, cognition, depressive symptoms, recent hospitalization, and survey wave/year. The recovery model was additionally adjusted for baseline ADL and IADL difficulty counts.

## Data Availability

The datasets analysed during the current study are available to registered users through the Health and Retirement Study data-access procedures [15–18], subject to the HRS conditions of use. HRS person-level data cannot be redistributed by the authors. Non-disclosive derived materials, harmonization documentation, and analytic code are available from the corresponding author on reasonable request, subject to the applicable data-use terms.

## Data Availability

The datasets analysed during the current study are available to registered users through the Health and Retirement Study (HRS) Data Portal (https://hrsdata.isr.umich.edu/), subject to the HRS registration requirements and Conditions of Use. The authors are not permitted to redistribute HRS person-level data.

## Abbreviations

ADL: activities of daily living
CES-D: Center for Epidemiologic Studies Depression scale
CI: confidence interval
HRS: Health and Retirement Study
IADL: instrumental activities of daily living
NIA: National Institute on Aging
RD: risk difference
SMD: standardized mean difference
